# Whole-genome sequencing reveals genetic diversity, population structure, and core collection construction in Korean peach (*Prunus persica*) germplasm

**DOI:** 10.3389/fpls.2025.1702527

**Published:** 2025-11-06

**Authors:** Seon-Hwa Bae, Namhee Jeong, Jung Hyun Kwon, Ju-Hyun Lee, Kidong Hwang, Youn Young Hur, So Jin Lee

**Affiliations:** 1 Fruit Research Division, National Institute of Horticultural and Herbal Science, Rural Development Administration, Jeonju, Republic of Korea; 2 International Technology Cooperation Center, Rural Development Administration, Jeonju, Republic of Korea; 3 Vegetable Research Division, National Institute of Horticultural and Herbal Science, Rural Development Administration, Jeonju, Republic of Korea

**Keywords:** *Prunus persica*, whole-genome sequencing, SNP, population structure, genetic diversity, core collection

## Abstract

Peach (*Prunus persica*) is an important temperate fruit crop and a model species for genomic research due to its diploid genome, short juvenile period, and relatively small genome size. Despite advances in next-generation sequencing (NGS), most peach genome-wide studies focused on a limited number of elite cultivars, and thus, the diversity of conserved germplasm is underrepresented. In Korea, a large number of peach genetic resources are maintained at the National Institute of Horticultural and Herbal Science (NIHHS), a branch of the Rural Development Administration (RDA), but no genome-scale core collection has been developed to date. This study aimed to perform whole-genome sequencing (WGS) on 445 peach accessions conserved in Korea between 2020 and 2025 using the Illumina NovaSeq 6000 platform, with the primary objective of constructing a representative genome-scale core collection and secondary objectives of identifying genome-wide single-nucleotide polymorphisms (SNPs) and assessing genetic diversity, population structure, and phylogenetic relationships. A total of 944,670 high-confidence SNPs were identified, with chromosomes 2 (G2) and 4 (G4) showing the highest variant density. Analyses using fastSTRUCTURE, principal component analysis (PCA), and phylogenetic reconstruction revealed a complex population structure and substantial genetic variation. From this data, a representative core collection was established, effectively capturing the majority of the genetic diversity present in the Korean peach germplasm. These results offer valuable genomic resources for peach improvement, marker development, pan-genome construction, and comparative genomics within the Rosaceae family.

## 1 Introduction

Peach (*Prunus persica* (L.) Batsch) is one of the most widely cultivated temperate fruit trees within the Rosaceae family, which also includes apple, cherry, and almond (Shulaev et al., 2008). Due to its diploid genome (2n = 2x = 16), relatively small genome size of 200~300 Mb (Debener and Linde, 2009; Shulaev et al., 2011; International Peach Genome Initiative et al., 2013; Meng and Finn, 2002), self-compatibility, and a short juvenile phase, peach has been established as a model species for genomic research on perennial fruit crops (Monet et al., 1996; Abbott et al., 2002). The domestication of peach dates back over 4,000 years in China (Aranzana et al., 2010), and since then the species has adapted to diverse climates and cultivation systems worldwide (Cao et al., 2014). Fossil evidence suggests that fruit edibility may have evolved even before domestication, which highlights a long and complex history of selection (Yu et al., 2018). Despite its global importance, genome-wide SNP-based studies on Korean peach germplasm have been limited. This shortcoming underscores the need for large-scale genomic characterization to support breeding, conservation, and the development of representative core collections.

Genomic studies of peach began in the early 1990s with the development of genetic linkage maps (Belthoff et al., 1992; Chaparro et al., 1994). The peach reference genome version 1.0 was first published in 2013 using a dihaploid genotype of the cultivar ‘Lovell’, which covers ~227.3 Mb and comprises 27,852 annotated genes (International Peach Genome Initiative et al., 2013). In 2017, the reference genome was updated to version 2.0 with improved sequencing depth and coverage of 99.2%, providing a more accurate foundation for genomic research (Verde et al., 2017). Earlier genetic mapping efforts, such as by Wang et al. (2002), laid the groundwork by determining that the *evergrowing* gene controlled shoot dormancy in peach. More recently, advanced studies like that of Fan et al. (2025) integrated whole-genome re-sequencing with machine learning techniques to refine quantitative trait locus (QTL) mapping for fruit quality traits, thus showcasing the expanding utility of genomic tools for trait dissection and breeding applications.

In China and Europe, studies have assessed the genetic diversity of peach accessions using not only SSRs and SNPs (Aranzana et al., 2010; Li et al., 2013; Vodiasova et al., 2025) but also other marker systems, including RAPDs (Cheng, 2007), retrotransposons, and iPBS markers (Naeem et al., 2021), and ISSRs (Demirel et al., 2024). In addition, gene-based and transposon-associated markers have recently been applied for trait-specific analyses in peach, such as skin color variation (Guo et al., 2023). These studies provide a foundation for breeding and conservation strategies and demonstrate the utility of diverse marker systems in peach germplasm analyses. However, they also highlight that SNP-based genome-wide studies are relatively scarce, particularly in underrepresented national collections, such as those in Korea.

Recent advances in next-generation sequencing (NGS) technologies have enabled genome-wide analyses of diversity, domestication history, and marker–trait associations (Ahmad et al., 2011). Large-scale initiatives have highlighted the transformative potential of genome-wide approaches, such as the global peach pangenome (Li et al., 2025), structural variation studies linked to malate content (Chen et al., 2025), and the PeachSNP170K array enabling population-scale trait analysis (Xu et al., 2025). Candidate genes such as *Prupe.6G290900* (OVATE family protein) and *Prupe.4G187100* (NAC transcription factor) have been associated with fruit traits and applied in breeding programs (Guan et al., 2021; Cao et al., 2023). However, because most studies have focused on a limited set of elite cultivars or geographically narrow collections, broader genetic diversity remains underexplored.

In Korea, core collections have been established for apple, pear, and grape (Kim et al., 2019; Chen et al., 2025; Bianchi et al., 2020). Although some peach diversity studies have been conducted (Hong et al., 2013), no genome-scale core collection has yet been developed using whole-genome sequencing (WGS). Given the current status of germplasm conservation, establishing a representative core set using WGS data is imperative to capture the genetic diversity of the Korean peach germplasm.

The establishment of a genome-based core collection that maximizes diversity while minimizing redundancy is crucial to enable the efficient management and utilization of peach genetic resources. Such collections serve as ideal populations for genome-wide association study (GWAS), enable the discovery of trait-associated genes, and function as cost-effective resources for marker development and breeding applications. For example, GWAS using national peach collections have already identified significant SNP–trait associations for traits like fruit skin hairiness (trichome presence) and flesh texture (Mas-Gómez et al., 2022). Moreover, the development of multisite reference collections, such as PeachRefPop across European countries, demonstrates the value of well-designed core sets for conserving and utilizing genetic resources under diverse environmental conditions (Cirilli et al., 2020).

Although significant progress has been made in peach genomics, the majority of studies have focused on elite cultivars or limited germplasm populations, restricting understanding of genome-wide diversity. In Korea, many peach accessions are conserved at the National Institute of Horticultural and Herbal Science (NIHHS), but no genome-based core collection has been established to date. However, the establishment of such a collection is essential to capture genetic diversity, reduce redundancy, and provide a foundation for molecular breeding, trait discovery, and long-term conservation. Therefore, we performed whole-genome sequencing on 445 Korean peach accessions to assess diversity, population structure, and construct a representative core collection, delivering genomic resources for peach improvement and sustainable utilization.

## 2 Materials and methods

### 2.1 Plant materials, genomic DNA extraction

**Figure 1:**
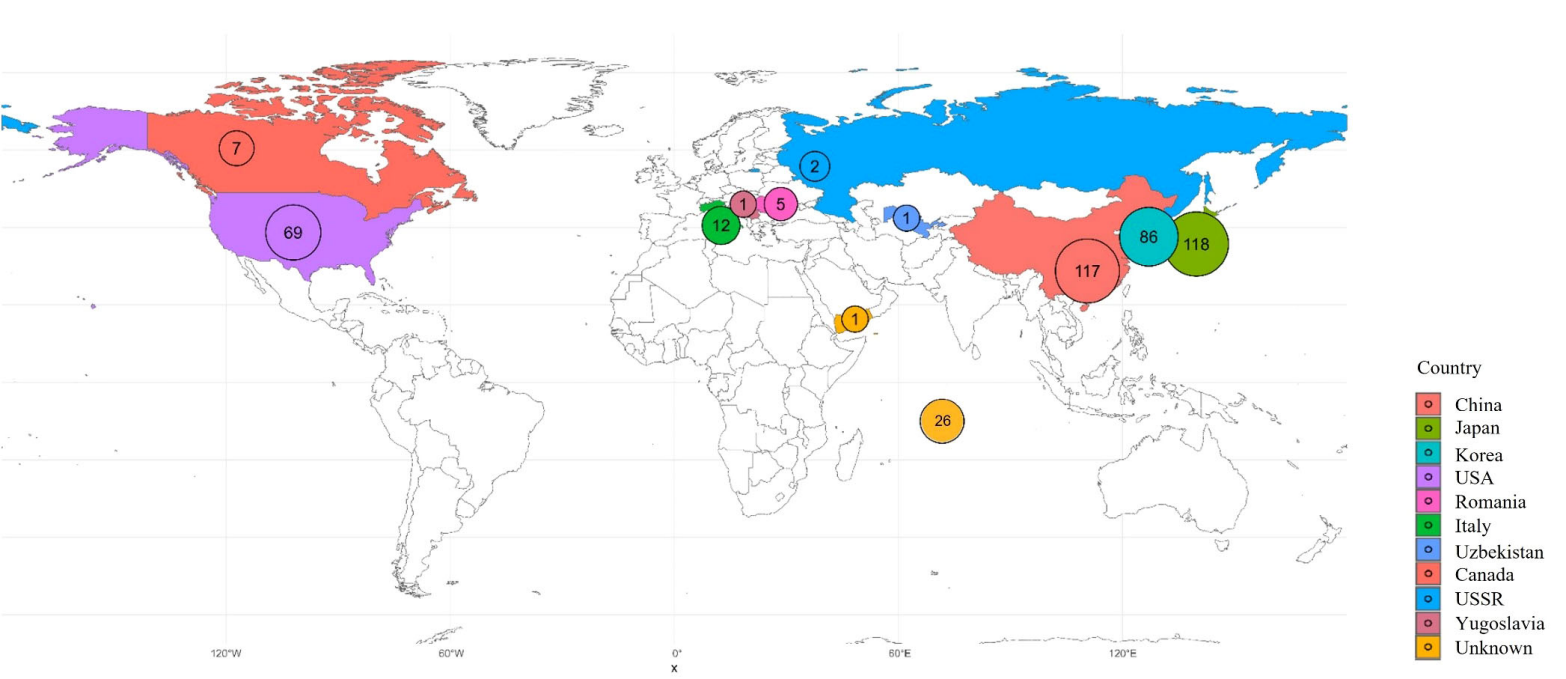
Map of the geographic distribution of the 445 samples collected. Sample counts per country are labeled on the map and color-coded as indicated.

A total of 445 peach accessions conserved at the National Institute of Horticultural and Herbal Science, Rural Development Administration (RDA), Republic of Korea, were used for this study. This collection includes a diverse set of Korean landraces, domestically bred cultivars, and accessions introduced from abroad. Information on countries of origin is summarized in **Figure 1**, and detailed accession data is provided in **Supplementary Table S1**. The study was carried out between 2020 and 2025.

Young leaves were collected in the field during the fully expanded leaf stage. Genomic DNA (gDNA) was isolated from young leaves using the Cetyltrimethyl ammonium bromide (CTAB) method (Doyle, J. and Doyle, 1987). The concentration and purity of extracted gDNA were assessed using a spectrophotometer (NanoDrop 1000, Thermo Scientific, USA), according to the manufacturer’s instructions, and the DNA was subsequently stored at −20 °C until required for WGS.

### 2.2 Whole-genome sequencing

DNA libraries were prepared from the gDNA using the TruSeq DNA Nano 550 bp Kit, according to the manufacturer’s instructions (Head et al., 2014). The prepared libraries had an insert size of ~550 bp. The libraries were then sequenced on the Illumina NovaSeq 6000 platform (Modi et al., 2021). Paired-end sequencing was performed with a read length of 2 x 151 bp at an average depth of 30× to ensure high-quality coverage. Raw reads were subjected to quality control using FastQC (Andrews, 2010) to evaluate read quality, GC content, and adapter contamination. A summary report was later generated using MultiQC (Ewels et al., 2016). The average Phred quality score was >34, and >96% of the bases had a quality score of ≥Q30. Adapter sequences and low-quality bases were trimmed with Trimmomatic (Bolger et al., 2014), and reads shorter than 36 bp were discarded. Only the quality-filtered reads were used for all downstream analyses.

### 2.3 Genomic alignment and variant calling

The quality-filtered reads were aligned to the peach reference genome (*Prunus persica* v2.0) using BWA-MEM v0.7.17 (Li and Durbin, 2009). Duplicate reads were marked and removed with Picard Tools (http://broadinstitute.github.io/picard/). Variant calling was then performed using the Genome Analysis Toolkit (GATK) v4.2.0.0 (McKenna et al., 2010). Subsequently, SNPs were identified using the GATK HaplotypeCaller (DePristo et al., 2011), which generated gVCF files for each sample to retain variant information for joint genotyping. These gVCF files were consolidated into a unified database with GenomicsDBImport, and joint genotyping was carried out across all samples.

For variant extraction, the initial variant call format (VCF) files were first assessed with BCFtools stats to obtain summary statistics and examine the distribution of SNPs indels, and other variants. SNPs and indels were separated into distinct files using GATK SelectVariants, and SNPs were further filtered with GATK VariantFiltration. Additional filtering was performed with VCFtools to assess the number and distribution of SNPs under different missing data thresholds (15%, 30%, 50%, and 75%) and minor allele frequency (MAF) cutoffs (5%, 10%, or 20%). Based on these results, the final SNP set was obtained by applying a missing data rate threshold of 30% and a MAF cutoff of 5%.

Two SNP genotype files were then generated: one of all filtered SNPs and another of all filtered SNPs with heterozygous SNPs removed. All final SNPs were annotated using SnpEff (v4.3t, http://snpeff.sourceforge.net/SnpEff.html#intro) to predict their functional impacts. These annotations included variants in genic, intergenic, and upstream/downstream regions along with their potential effects (e.g., synonymous, missense, high-impact) (Cingolani et al., 2012).

### 2.4 Genetic structure, diversity, and phylogenetic analysis

Bayesian population structure was analyzed using the fastSTRUCTURE program (Raj et al., 2014), based on a high-quality SNP dataset. The number of genetic clusters (*K*) ranged from 1 to 10, and the analysis was performed in simple mode with 10 cross-validation folds (“–cv=10”). The optimal *K* value was determined using the choose.py script, which identifies the model complexity that maximizes marginal likelihood while minimizing redundancy in explaining population structure. Results were visualized using the StructureSelector tool (Puechmaille, 2016).

Principal component analysis (PCA) was conducted using a filtered set of high-quality SNP loci to determine genetic diversity among the 445 peach accessions. Multivariate analyses were performed with the R package adegenet (Jombart and Ahmed, 2011), during which PCA was used to summarize genetic variation among individuals. Additionally, discriminant analysis of principal components (DAPC) was applied to describe the population structure further and to visualize the genetic relationships among samples.

Phylogenetic analysis was performed using the final set of SNP loci selected after filtering. In addition, a Maximum Likelihood (ML) tree was inferred using MEGA X (Kumar et al., 2018), utilizing 1,000 bootstrap replicates. Both phylogenetic trees were visualized as unrooted circular trees using the R package ape (Paradis and Schliep, 2018).

### 2.5 Construction of core collection

A core collection was constructed using a previously described systematic procedure to eliminate genetic redundancy and maximize representativeness (Osorio-Guarin et al., 2025). First, genetic similarity between all samples was evaluated to identify duplicates or closely related accessions. Samples with a genetic similarity (PI_HAT) of ≥ 95% were considered duplicates; this process was facilitated using the poppr package in R (Kamvar et al., 2014). After identifying these duplicate groups, a single representative sample was selected from each. The selection criteria prioritized samples with the lowest missing data rate, complete phenotypic information, a clear origin, and lineage data.

The final core collection was constructed using Genocore analysis (Jeong et al., 2017), which was used to generate candidate core collections of accessions that represented over 99% of the total genetic diversity. The final list was then reviewed and manually adjusted by peach breeding experts to ensure the final core collection was both efficient and genetically diverse.

## 3 Results

### 3.1 Whole-genome sequencing and SNP discovery

Whole-genome re-sequencing was performed on the 445 peach accessions, including wild-type individuals, using the Illumina NovaSeq 6000 platform, which generated high-quality paired-end reads with an average sequencing depth of 30×. A total of 3,441,542 SNPs were initially identified, and after filtering (MAF ≥ 0.05; missing data ≤ 30%), 944,670 high-quality SNPs were retained for analyses (**Supplementary Table S2**).

### 3.2 Genome-wide distribution and functional annotation of variants

**Figure 2:**
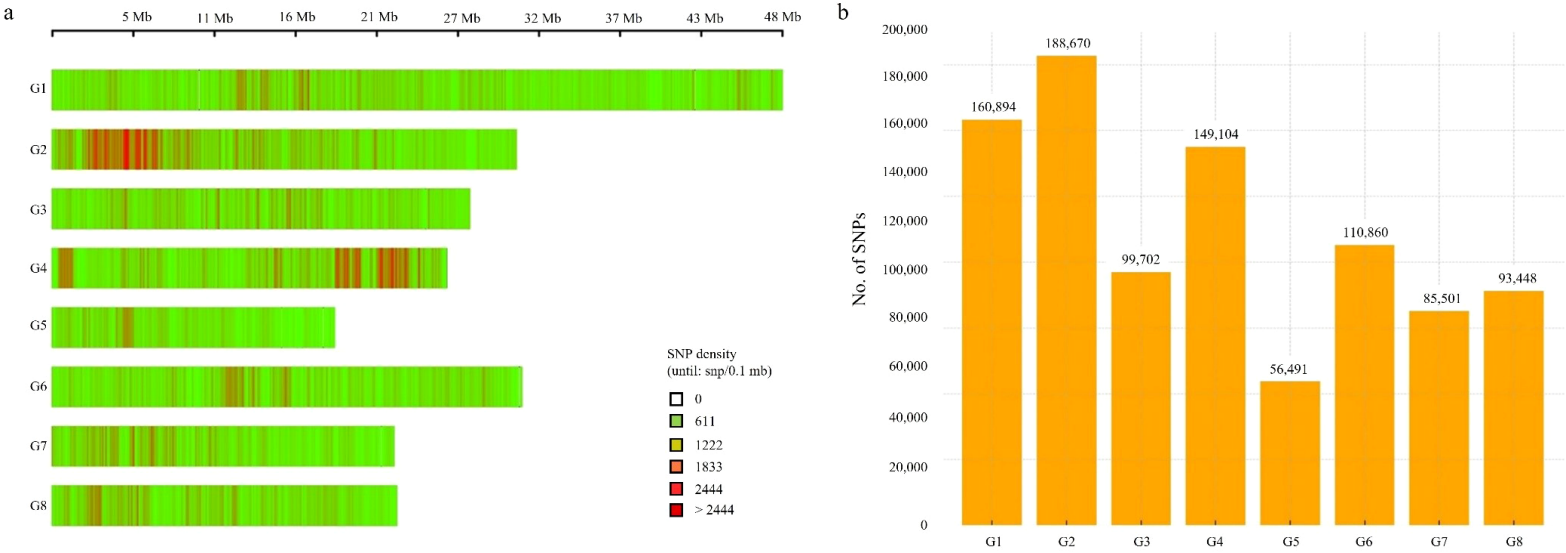
Chromosomal distribution of SNPs in the peach genome. **(a)** SNP density per 100 kb window across eight chromosomes (G1–G8). Color intensities indicate SNP density within each window: light green denotes low SNP density, while orange to deep red indicates high density. **(b)** Total number of filtered SNPs per chromosome (n = 944,670).

The 944,670 high-quality SNPs were mapped onto the eight chromosomes of the peach genome. Their distribution was visualized based on SNP density calculated per 100 kb window (**Figure 2a**). The SNP density plot revealed that SNPs were unevenly distributed across the genome, with regions of relatively high and low variation. Notably, chromosomes G2 and G4 exhibited the highest SNP densities, with several regions exceeding 2,444 SNPs per 100 kb (indicated by red bars). In particular, SNP hotspots were observed around the 5 Mb region of G2 and the 22 Mb region of G4, where continuous high-density SNP clusters, each containing > 2,332 SNPs per 100 kb, were identified. In contrast, chromosomes G5 and G7 showed comparatively few high-density regions. This distribution pattern provides a basis for identifying genomic regions with concentrated polymorphisms, which may be valuable in future genetic studies. In addition, the chromosomal distribution of 944,670 filtered SNPs was examined (**Figure 2b**). Chromosome G2 harbored the highest number of SNPs (188,670), followed by G1 (160,894) and G4 (149,104). On the other hand, chromosome G5 had the fewest SNPs (56,491). These results indicate that SNP and polymorphism densities vary significantly among chromosomes and provide important insight for genomic diversity assessments and the selection of target regions for further analysis.

A comprehensive analysis of SNPs and Indels was conducted to evaluate the broader functional landscape of genomic variation. Functional annotation using SnpEff v4.3t categorized SNPs into four impact levels: modifier (2,323,492), low (93,028), moderate (55,301), and high (1,819). Notably, a significant proportion of these SNPs were located within or adjacent to genes involved in stress response and developmental regulation. Among these, NBS-LRR resistance gene families exhibited a particularly high density of functional variants, suggesting that these regions may serve as adaptive hotspots associated with disease resistance and environmental fitness (**Table 1**).

**Table 1:** Classification of variants by their predicted effects using SnpEff.

Impact of SNP effect	SNP effect	No.
Low	Splice_region_variant	6,502
	5_prime_UTR_premature_start_codon_gain_variant	971
	Stop_retained_variant	63
	Synonymous_variant	42,746
	Sum	50,282
Modifier	Intergenic region	695,169
	Upstream_gene_variant	691,026
	Downstream_gene_variant	634,043
	Intron variant	212,254
	3_prime_UTR_variant	32,209
	Intragenic_variant	32,945
	5_prime_UTR_variant	24,689
	Non_coding_transcript_exon_variant	1,157
	Sum	2,323,492
Moderate	Missense_variant	55,301
High	Splice_donor_variant	150
	Splice_acceptor_variant	182
	Stop_gained	924
	Stop_lost	152
	Start_lost	79
	SPLICE_SITE_ACCEPTOR	182
	SPLICE_SITE_DONOR	150
	Sum	1,819

### 3.3 Population structure and genetic relationships

**Figure 3:**
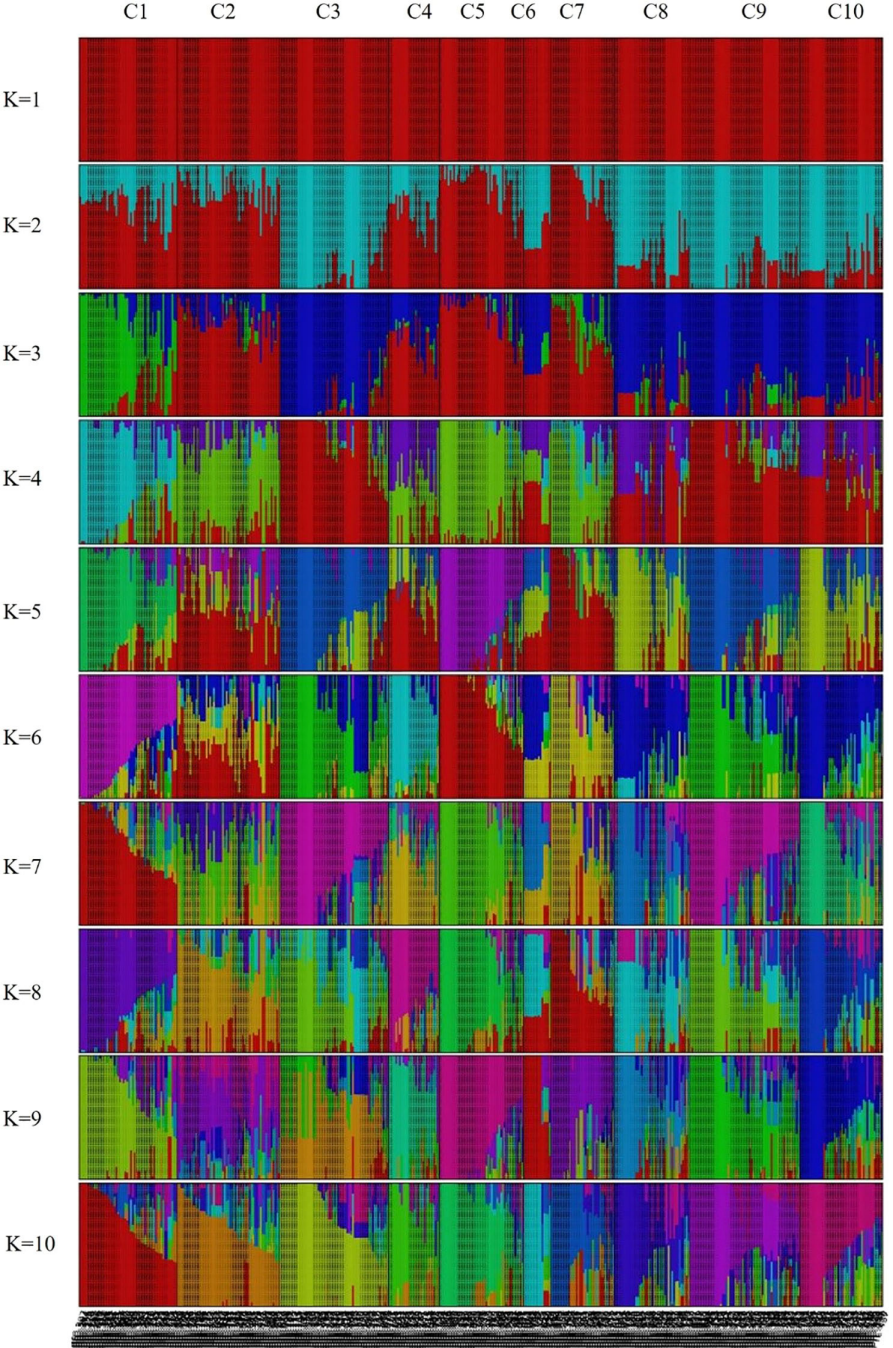
Population structure of the 445 *Prunus persica* accessions inferred using fastSTRUCTURE (*K* = 1–10). Each vertical bar represents an accession, and colors indicate the proportion of genetic membership in each cluster.

Population genetic structure analysis was performed using 944,670 SNPs derived from the 445 peach accessions. The optimal number of genetic clusters (*K*) was estimated based on genome-wide genotype data using cross-validation error (CV error) and marginal likelihood values. As a result, *K* = 10 was determined to be the most appropriate cluster number (**Supplementary Figure S1**), as it had the highest likelihood score (–0.857) and the lowest CV error (0.373). Accordingly, the 445 accessions were classified into ten groups (**Figure 3**). Visualization of the Q matrix at *K* = 10 revealed complex patterns of population differentiation and genetic admixture, indicating the presence of multiple ancestral lineages within the genetic resource collection (**Figure 3**). However, the effective number of model components, calculated from mean Q values across clusters, was close to one, indicating that most accessions were assigned mainly to a single ancestral cluster with limited admixture.

**Figure 4:**
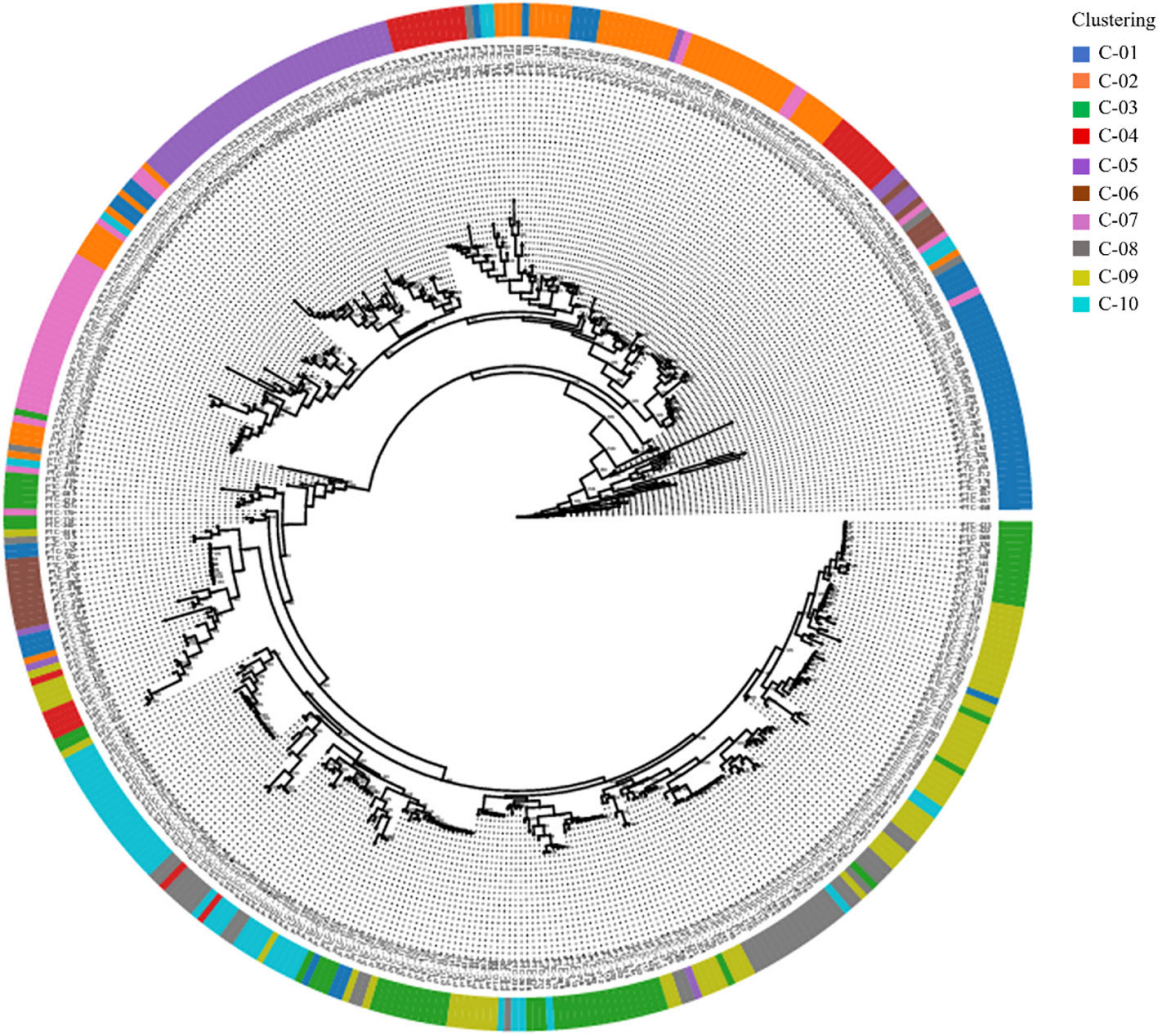
The phylogenetic relationships of the 445 peach accessions. Maximum-likelihood tree with bootstrap values. The outer circle represents 10 clusters with structure results; different colors indicate different clusters.

A phylogenetic tree was constructed using the Maximum Likelihood (ML) method, based on the ten genetic clusters identified in **Figure 3**, to explore the evolutionary relationships among clusters (**Figure 4**). The analysis included 445 *Prunus persica* accessions, including the wild-type accession PHC-458, which was designated as the outgroup. Non-informative positions were excluded from each sequence pair, resulting in a final alignment of 906,754 positions. The resulting tree showed that most clusters resolved into distinct monophyletic groups, often comprising one or a small number of lineages. In particular, the purple (C05), blue (C01), and pink (C07) clusters occupied relatively large and compact segments of the tree, although C01 was split into more than one adjacent clade. By contrast, the orange (C02), green (C03), olive (C09), and gray (C08) clusters were widely dispersed across multiple branches, reflecting admixed ancestries and polyphyletic origins. However, in some clusters, the accessions were dispersed across multiple branches, suggesting that these clusters are genetically polyphyletic and composed of individuals with diverse ancestral origins. Pairwise population differentiation among the ten clusters was also evaluated (**Supplementary Table 3**). Average pairwise *F_ST_* (0.088) and Φ_ST (0.147) values indicated relatively low genetic divergence among clusters, suggesting that most accessions share a common genetic background despite their subdivision.

### 3.4 Principal component analysis of genetic diversity and representativeness of the core collection

**Figure 5:**
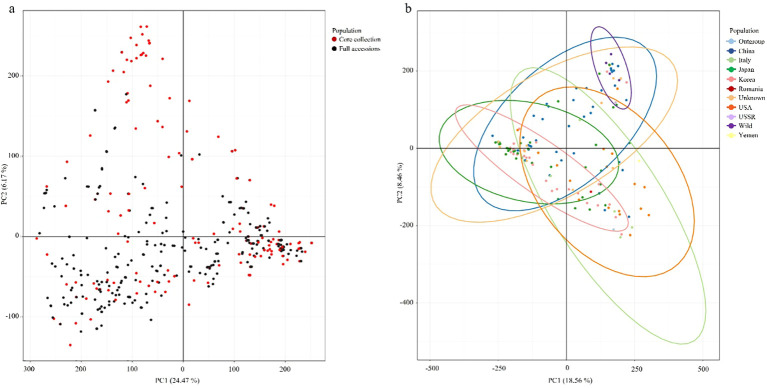
Principal component analysis (PCA) of peach accessions. **(a)** PCA of all 445 peach accessions (black dots), with the 150 core collection accessions highlighted in red. **(b)** PCA of the 150 core collection accessions grouped by country of origin with colors indicating the country of origin.

PCA was used to evaluate the genetic diversity of the 445 peach accessions and assess the representativeness of the Core collection. By considering genetic representativeness and redundancy elimination, a core collection consisting of 150 accessions (Core150) was constructed. Based on 944,670 high-quality SNPs, PCA of the entire population revealed that PC1 and PC2 explained 24.47% and 16.17% of the total genetic variance, respectively (**Figure 5a**). The Core150 accessions (red dots) were broadly distributed across the genetic spectrum of the full collection (black dots), indicating that these accessions effectively captured overall genetic diversity. An independent PCA was also conducted using the Core150 accessions, based on 944,670 SNPs, with samples categorized by country of origin (**Figure 5b**). In this analysis, PC1 and PC2 accounted for 18.56% and 18.46% of the variance, respectively, with PC1 explaining the most significant variation. The Core150 accessions showed an even distribution across PCA space, without bias toward any specific geographic region, suggesting that the core collection reflected broad geographic origins. These results confirm that the Core150 subset represents the genetic structure of the entire genetic resource collection and is well-suited for downstream applications such as genome-wide association studies (GWAS), marker development, and the conservation of genetic resources.

## 4 Discussion

In this study, we conducted a comprehensive population genetic analysis of 445 diverse *Prunus persica* accessions using whole-genome re-sequencing data that yielded 944,670 high-quality SNPs. fastSTRUCTURE analysis revealed that *K* = 10 was the optimal number of clusters (**Figure 3**), highlighting the highly stratified and diverse genetic landscape of the peach germplasm. Unlike earlier studies that which relied on SSR (Aranzana et al., 2010), ISSR (Demirel et al., 2024), RAPD (Cheng, 2007), or retrotransposon-based iPBS markers (Naeem et al., 2021), which analyzed smaller sample sets and employed low-density marker systems typically resolving only a few major groups, our genome-wide high-density SNP dataset provided a substantially higher resolution for population inference. By analyzing 944,670 loci across 445 Prunus persica accessions, this study identified finer-scale genetic structures and revealed more complex patterns of population differentiation than previously observed. The consistent clustering results obtained from fastSTRUCTURE, PCA, and phylogenetic analyses (**Figures 3**-**5**) suggest that genome-wide SNP data can detect subtle intra-group variation and potential sublineages within groups that appeared homogeneous in earlier studies (Guo et al., 2023). These findings collectively imply that genome-wide SNP analysis offers a more powerful framework to interpret the evolutionary relationships and diversification history of Prunus persica, complementing and extending the insights from previous SSR- or retrotransposon-based studies. This high-resolution approach enables a deeper understanding of the domestication history and population differentiation of peach germplasm, as supported by recent genome-wide analyses (Cao et al., 2014; Micheletti et al., 2015; Shi et al., 2020; Yu et al., 2018).

This study revealed a relatively low average pairwise *F_ST_* (0.088) among clusters (**Supplementary Table 3**), reflecting weak differentiation within cultivated accessions, which is consistent with earlier reports of a narrow founder base and a historical genetic bottleneck in peach (Aranzana et al., 2010). In contrast, genome-wide studies including wild relatives and landraces have reported higher *F_ST_* values (Yu et al., 2018), underscoring the importance of broadening the genetic base and introducing novel alleles from diverse germplasm for future breeding. The phylogenetic tree further supported PCA and fastSTRUCTURE results and also revealed polyphyletic clusters (Martínez-García et al., 2013; Cao et al., 2014), suggesting contributions from multiple ancestral lineages. Together, these findings highlight the complex domestication and breeding history of *Prunus persica* and the need for integrative approaches that combine clustering and phylogenetic perspectives.

Beyond resolving population structure, this study established a genetically diverse and non-redundant core collection (Core150), which retained >99% of nucleotide diversity while maintaining balanced representation across geographic origins and genetic groups. The establishment of Core150 demonstrates its value not only as a representative subset but also as a practical panel for downstream applications. Specifically, Core150 provides an ideal GWAS population (Cao et al., 2016), a robust resource for marker-assisted selection, and a tool for conservation prioritization by preserving rare alleles and unique genetic backgrounds. Similar strategies in other fruit crops have shown that well-designed core collections effectively capture genetic diversity while minimizing redundancy (Brown, 1989; Odong et al., 2011). Thus, Core150 bridges the gap between population genomics and applied breeding, ensuring both representativeness and practical utility.

This analysis was anchored to the updated peach reference genome (Verde et al., 2017), which provides a robust foundation for variant interpretation and gene annotation. Leveraging this framework, our study also identified SNP-rich chromosomal regions (“hotspots”), which may represent genomic regions under selection or harbor loci associated with adaptive or agronomic differentiation within the peach population. Previous studies have demonstrated that genes located within such SNP-dense regions are functionally associated with important agronomic traits, including disease resistance and stress adaptation, in crops such as peach (Da Silva Linge et al., 2022; Li et al., 2021), olive (Bazakos et al., 2023), potato (Sharma et al., 2024), *Brassica juncea* (Sudan et al., 2019), and sorghum (Getahun et al., 2025). These findings suggest that some of the hotspot regions identified in this study may also harbor functionally important genes that influence stress responses or resistance-related pathways, and thereby provide potential targets for molecular breeding in peach. However, high-impact SNPs and hotspot regions were not functionally validated, which constitutes a study limitation. Future strategies such as transcriptomic validation, expression profiling, and candidate gene–trait association will be essential to confirm their biological roles and translate genomic signals into practical breeding markers. Such follow-up efforts will also help disentangle causal variants from linked polymorphisms and thereby improve the precision of molecular breeding.

In conclusion, high-resolution fastSTRUCTURE and PCA analyses presented in this study, together with the establishment of the Core150 collection, refine and extend the insights provided by previous marker-based studies. By combining genome-wide diversity coverage with practical applications, Core150 provides a cornerstone framework for GWAS, marker-assisted breeding, and conservation strategies. Moving forward, the integration of phenotypic and environmental data with expanded global germplasm will further strengthen the role of Core150 as a valuable resource for peach improvement and long-term genetic resource management.

## 5 Conclusions

In this study, we performed whole-genome sequencing of 445 peach accessions conserved in Korea and established a core collection of 150 accessions (Core150) that captured over 99% of total nucleotide diversity. Core150 enables effective management and diversity assessment of peach genetic resources and serves as a foundation for GWAS, marker development, and breeding applications when integrated with phenotypic data. Moreover, the genomic resources generated here serve as a critical foundation for comparative and applied genomics within the Rosaceae family and have practical applications in peach breeding and conservation.

## Data Availability

The raw sequencing reads generated in this study have been deposited in the NCBI Sequence Read Archive (SRA) under BioProject ID [PRJNA1328824].
