# Supplementary material for: Whole-genome sequencing reveals genetic diversity, population structure, and core collection construction in Korean peach (Prunus persica) germplasm
Source: Front Plant Sci. 2025 Nov 6;16:1702527. doi: 10.3389/fpls.2025.1702527 (PMC12631232; doi:10.3389/fpls.2025.1702527)
Supplement: Supplementary file 1 [file Table1.docx]

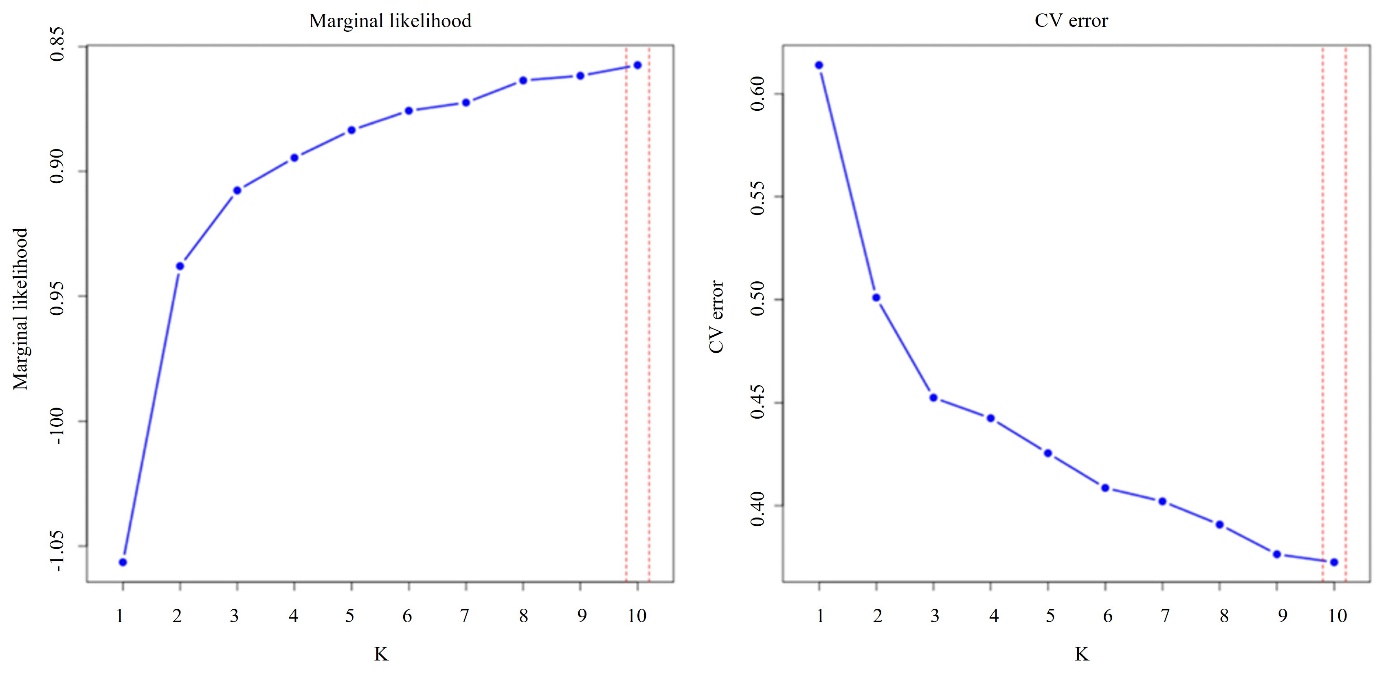


Figure S1. Cross-validation error and marginal likelihood values were calculated for each K value to estimate the optimal number of genetic clusters. Both methods indicated that K = 10 provides the best fit for the population structure.
